# The effect of supplementing pre-weaning dairy calves’ starter feed with *Nigella sativa* and sesame meals on growth performance, blood metabolites, immunity, and health

**DOI:** 10.5455/javar.2026.m1028

**Published:** 2026-03-24

**Authors:** Vahid Fadaei Muloodi, Kazem Karimi, Jafar Fakhraei, Naser Karimi, Ghobad Asgari Jafarabadi

**Affiliations:** 1 Department of Animal Science, Varamin-Pishva Branch, Islamic Azad University, Varamin, Iran

**Keywords:** Blood parameters, feed efficiency, growth, health, immunity, pre-weaning calf

## Abstract

**Objectives:** This study investigated the effects of dietary inclusion of *Nigella sativa* meal (NSM) and sesame meal (SM) in the starter feed on growth performance, blood metabolites, immunity, and health of pre-weaning Holstein calves.

**Materials and Methods:** Forty male calves were used in 2 × 2 factorial arrangements in a completely randomized design. The four treatments were: 1) Control (basal diet), 2) Basal diet + 5% NSM, 3) Basal diet + 5% SM, and 4) Basal diet + an equal mixture of NSM and SM (NSM × SM).

**Results:** Results indicated that treatments containing NSM and SM significantly (*p* < 0.05) improved growth performance, feed intake, immunity, and health. The NSM × SM interaction increased final body weight gain by 7.66%, 10.53%, and 7.32% compared to the control, NSM, and SM treatments, respectively. This treatment also superiorly improved feed efficiency by 16.67%, 12.35%, and 7.06%, respectively. The NSM × SM group showed the lowest serum concentrations of glucose (19% lower), triglycerides (19.5% lower), and cholesterol (14.2% lower) than the control. Conversely, it had the highest serum concentrations of total protein (11% higher), albumin (17.9% higher), immunoglobulin G (IgG, 36.9% higher), and white blood cell (WBC) count (33.1% higher) compared to the control.

**Conclusions:** In conclusion, incorporating 5% sesame and *N. sativa* meals, especially as an equal mixture, into calves’ starter feed enhanced growth rate, feed efficiency, immunity, and overall health in pre-weaning dairy calves.

## 1. Introduction

The pre-weaning period is considered one of the most critical stages in raising dairy calves, as it plays a vital role in their growth, development, and health. From an economic perspective, extending the pre-weaning period is not cost-effective. Therefore, providing appropriate nutrition while simultaneously focusing on physical growth and the development of the gastrointestinal tract, with the aim of accelerating the initiation of nutrient fermentation, the production of volatile fatty acids and microbial protein at adequate levels, ensuring gastrointestinal health, and ultimately improving immunity and overall calf health, are among the most important management practices during this period [1, 2]. Pre-weaning calves, due to their rapid growth and organ development, require diets with high nutrient density. Such diets play a crucial role in the formation of the immune system, skeletal and muscular growth, and gastrointestinal development. Any nutritional deficiency during this period can have long-term negative effects on the calf’s growth and health [3, 4]. The relationship between the quantity and quality of calf nutrition and growth performance, feed efficiency, and improvements in immunity and health at weaning and subsequent stages has been well established [5, 6, 7].

The use of phytogenic compounds in livestock diets has garnered significant attention in recent years due to their bioactive properties, including antioxidants, anti-inflammatory agents, and immune and health enhancers [2, 8]. Numerous studies indicate that supplementing pre-weaning calves’ diets with these meals increases body weight and improves immunity and health. This improvement in growth performance is attributed to enhanced nutrient absorption and appetite stimulation [9, 10, 11]. *Nigella sativa* (black cumin) and sesame meals, owing to their valuable phytogenic compounds such as thymoquinone, thymol, sesamin, and sesamolin, improve gastrointestinal health by reducing oxidative stress and inflammation in pre-weaning calves, prevent the occurrence of infectious diseases, and thus possess high potential for enhancing growth, overall health, and immunity in animals [11, 12, 13]. Furthermore, sesame and *N. sativa* meals contain valuable nutritional compounds that can be utilized to complement the characteristics of calf diets [2, 14]. Sesame and *N. sativa* meals are by-products of oil extraction from their respective seeds and contain significant amounts of protein, essential amino acids, vitamins, and minerals [10, 15]. These compounds can improve growth and organ development in calves.

Moreover, in addition to the aforementioned nutrients, these meals possess anti-inflammatory and antioxidant properties due to the bioactive compounds mentioned above [16, 17], which can help strengthen the immune system and improve calf health, exerting positive effects on growth performance and health indices [8, 14]. Therefore, the use of high-quality natural feed resources in the starter diet can largely mitigate the aforementioned challenges and improve calf growth and health [1, 4, 18].

Improved growth performance and feed efficiency, along with enhanced blood parameters and health status, are key indicators for evaluating pre-weaning calf diets [12, 19, 20]. Consequently, investigating the effect of dietary sesame and *N. sativa* meals on the aforementioned indices can provide valuable information. While the nutritional and health effects of these meals and their interactions have been largely elucidated in mature ruminants, their effects in pre-weaning calves remain ambiguous, and research gaps remain. The present study was conducted to investigate the effects of dietary inclusion of different levels of *N. sativa* and sesame meals independently or in combination mixed with starter feed on growth performance, feed intake, blood metabolites, immunity, and health of pre-weaning Holstein calves.

## 2. Materials and Methods

### 2.1. Ethical approval

All procedures used on the animals in this study were approved by the Islamic Azad University Institutional Animal Care and Use Committee (reference number: IAUEC 2023/1402-423-36-27).

### 2.2. Animals and experimental design

A 65-day experiment was conducted from March to May 2023 at a large commercial dairy farm (Fadaei Agriculture and Industry Co., Saveh, Iran). Forty male Holstein calves with an average initial body weight of 42.38 ± 3.08 kg and age of 3 ± 0.5 days were used. From birth until weaning at 65 days, calves were randomly assigned to one of four dietary treatments (*n* = 10 per treatment). The experimental diets were as follows: (1) Control: Basal starter diet. (2) NSM: Basal diet supplemented with 5% *Nigella sativa* meal. (3) SM: Basal diet supplemented with 5% sesame meal. (4) NSM × SM: basal diet supplemented with an equal mixture of the two meals (total 5%).

The measured parameters included weight gain, dry matter intake, feed efficiency, biochemical blood parameters, immunity, and health traits throughout the experimental period.

### 2.3. Management and recording

Calves were weighed at birth and at weaning, in accordance with the necessary protocols. Their precise weight was recorded using a digital scale with an accuracy of 50 gm, and the average weight gain during the specified intervals was calculated. Prior to the experiment, all calves underwent a thorough veterinary examination. Their specifications were recorded, followed by precise weighing. Colostrum feeding was administered completely to all calves for the first 3 days after birth at a rate of 4 L (approximately 10% of body weight) in two feedings per day. The thermal humidity index (THI) during the experiment ranged from 70 to 85. After calving, the cows were housed in individual calving stalls for 48 h; afterwards, they were moved to postpartum stalls for 65 days. Calves were randomly housed individually in holding places with straw bedding (1.8 × 2.0 m) and were kept under standard hygienic conditions. After placement in their pens, calves had free access to clean water and a weighed amount of feed. Furthermore, fresh milk containing 12% dry matter was provided twice daily at 0600 and 1800 h. Daily milk intake was similar for all experimental groups: on average, 4 kg/day during the first 30 days, 5–6 kg/day during the second month, and gradually reduced towards the end of the period, with complete cessation at 65 days of age. Free access to experimental diets was provided from 3 days of age, coinciding with the start of the trial. Daily feed intake was measured and recorded throughout the experimental period. The results of the chemical composition analysis of the feed ingredients are presented in Table 1.

**Table 1.** Chemical composition of the feed ingredients (%DM).

Nutrients	SBM	AH	SH	BF	CF	WB	NSM	SM
DM	97.51	91.00	95.90	89.58	90.21	91.70	94.03	93.69
CP	41.50	15.65	3.88	11.09	9.14	15.45	35.87	44.27
ME (mcal/kg)	3.78	2.54	2.23	3.52	3.90	2.55	3.54	4.06
EE	3.34	1.15	0.95	2.52	3.30	3.18	14.78	10.90
Ash	7.67	9.19	10.35	3.55	1.49	6.35	6.53	9.53
CF	9.50	29.75	38.72	66.70	3.34	14.52	6.88	5.06
Ca	0.67	1.55	0.39	0.15	0.12	0.15	1.06	2.63
P	0.72	0.22	0.09	0.18	0.09	0.35	1.07	1.36

DM: dry matter; CP: crude protein; ME: metabolizable energy; EE: ether extract; CF: crude fiber; Ca: Calcium; P: Phosphorus; SBM: Soybean meal; AH: Alfalfa hay; SH: Straw hay; BF: Barley flour; CF: Corn flour; WB: Wheat bran; NSM: *Nigella sativa* meal; SM: Sesame meal.

### 2.4. Feed sampling and analyses

Samples of the feedstuffs used in the diets were collected prior to the start of the experiment and sent to a specialized animal nutrition laboratory for chemical composition analysis. In the laboratory, samples were ground using a mill equipped with a 2-mm sieve for chemical analysis. Subsequently, dry matter (at 65°C for 48 h), crude protein (Kjeldahl method), crude fat (Soxhlet method), neutral detergent fiber (NDF) and acid detergent fiber (ADF) (Van Soest method), and ash (using an electric furnace at 550°C) were determined according to standard procedures recommended by AOAC [21]. Calcium and phosphorus content in the samples were also measured using atomic absorption spectrometry. All chemical analyses were performed in duplicate.

### 2.5. Feed formulation and experimental diets

The experimental diets were formulated based on the standard nutrient requirements of pre-weaning calves according to National Research Council [3] recommendations. using the AminoCow software version 2-5-3. The ingredients and chemical composition of the experimental diets are provided in Table 2.

**Table 2.** Ingredients and chemical composition of experimental diets.

Feed ingredients (%)	Control	NSM 5%	SM 5%	(NSM × SM) 5%
Alfalfa hay	5.10	5.00	5.00	5.00
Straw hay	5.10	5.00	5.00	5.00
Barley flour	15.90	15.80	15.80	15.80
Corn flour	37.82	37.19	35.64	35.75
Soybean meal 44%	24.78	22.80	22.25	22.15
Wheat bran	9.30	7.21	9.31	9.30
*Nigella sativa* meal	0	5.00	0	2.50
Sesame meal	0	0	5.00	2.50
DCP	0.60	0.60	0.60	0.60
Salt	0.20	0.20	0.20	0.20
Sodium Bicarbonate	0.20	0.20	0.20	0.20
Vitamin and Mineral Premix*	1.00	1.00	1.00	1.00
Chemical Composition of the diets (%)				
CP	21.75	21.32	21.41	21.45
NEm (mcal/kg)	1.95	1.94	1.95	19.4
NEg (mcal/kg)	1.31	1.30	1.31	1.30
NDF	22.11	21.66	21.41	21.79
ADF	11.11	10.78	10.71	10.91
Ash	7.22	7.57	7.87	7.59
EE	3.10	3.11	3.20	3.12
NFC	49.61	47.96	47.26	44.94
Ca	0.53	0.50	0.51	0.54
P	0.34	0.33	0.35	0.34

NSM: *Nigella sativa* meal; SM: Sesame meal; NSM × SM: *Nigella sativa* × sesame meal; DCP: dicalcium phosphate; CP: crude protein; NEm: net energy for maintenance; NEg: net energy for growth; NDF: neutral detergent fiber; ADF: acid detergent fiber; EE: ether extract; NFC: non-fiber carbohydrate; Ca: Calcium; P: Phosphorus. *Per kg of the premix contained: 250,000 IU vitamin A, 50,000 IU vitamin D, 1500 IU vitamin E, 120 gm Calcium, 20 gm Phosphorus, 20.50 gm Manganese, 186 gm Sodium, 2.25 gm Manganese, 7.70 gm Zinc, 1.25 gm Iron, 3 gm Sulfur, 14 gm Cobalt, 1.25 gm Copper, 56 gm Iodine, and 10 gm Selenium.

### 2.6. Blood sampling and analyses

Blood samples were collected from the jugular vein of calves at the conclusion of the experimental period (day 65), approximately 4 h after morning feeding. One aliquot per calf was used to measure red blood cell (RBC) and white blood cell (WBC) counts via a complete blood count (CBC) analyzer (Sysmex xp100). Enumeration and differentiation of red and white blood cells were performed using the differential staining method with a hemocytometer [10, 22]. Another aliquot was subsequently centrifuged at 3000× *g* for 15 min to isolate the serum, which was stored at –20°C until further processing. Blood biochemical parameters were analyzed using a CS-400 auto chemistry analyzer (Core Medical Co., Ltd., China) with Pars-Azmoon commercial kits (Iran; catalog numbers: glucose, 117500; triglyceride, 132500; cholesterol, 110500). All analyses were performed in duplicate. Before any samples were processed, the analyzer was calibrated with control serum containing known concentrations of N and P (TrueLabN^®^ and TrueLabP^®^, Pars Azmoon Co., Tehran, Iran) and a standard calibration solution (TrueCal U^®^, Pars Azmoon Co., Tehran, Iran).

### 2.7. Health characteristics and analyses

To evaluate health traits and fecal consistency (FC) in calves, a standardized health monitoring chart was employed [23, 24]. Daily recordings included clinical signs of health (such as fever, diarrhea, pneumonia, and other infectious diseases) and each calf’s fecal consistency. The time, date, and description of any procedures performed (veterinary examination and treatment) were meticulously documented in these forms. A general health score (GHS) for each calf was calculated using the following formula [23, 25]:


\[{\mathrm{GHS}} = 28 - \left[ {(2{\mathrm{\;}} \times {\mathrm{a}}) + (2{\mathrm{\;}} \times {\mathrm{b}}) + (2{\mathrm{\;}} \times {\mathrm{c}})} \right]\]

In this formula, the letters represent the following parameters: a = Number of days with fever; b = Number of diarrhea or digestive disorder events; c = Number of treatments for diarrhea, pneumonia, or other infectious diseases. Furthermore, fecal consistency was assessed according to the methodology introduced by Renaud et al. [26]. This classification system categorizes calf fecal consistency into four distinct groups, including: Group 1: Very firm consistency; Group 2: Moderately firm, normal consistency; Group 3: Soft or watery consistency; Group 4: Diarrheic feces, with the presence of mucus or blood. Therefore, according to this scoring system, the acceptable fecal status is defined within the score range of 2–3, with the optimal condition being a score of two [27].

### 2.8. Statistical analysis

Data obtained from this experiment were analyzed as a completely randomized design in a 2 × 2 factorial arrangement using a Mixed Model (Model 1) and the effect of initial calf weight as a covariate using SAS 9.4 software [28].

Model (1)—


\[{{\rm Y}_{\rm ijk}} = {\rm \mu} + {{\rm A}_{\rm i}} + {{\rm B}_{\rm j}} + {({\rm AB})_{{\rm ij}}} + {\rm \beta}\,({{\rm W}_{{\rm ijk}}} - \overline {\rm W} \ldots) + {{\rm e}_{{\rm ijk}}}\]

Components of Model (1) include: Y_ijk_ = an observation of the trait in question; μ = overall population mean of the trait. A_i_ = effect of the first factor (e.g., *Nigella sativa* meal), B_j_ = effect of the second factor (e.g., sesame meal), (AB)_ij_ = interaction effect between the first and second factors, \[{\rm \beta}\,({{\rm W}_{{\rm ijk}}} - \overline {\rm W}\ldots)\] = effect of initial calf weight as a covariate, and e_ijk_ = experimental error. The period/time effect and the experimental error were considered random effects, while all other factors were considered fixed. The Shapiro–Wilk test was also used to assess the normality of the data. Mean comparisons among treatments were performed using Duncan’s test at a significant level of less than 5% (*p* < 0.05).

## 3. Results and Discussion

### 3.1. Weight gain, dry matter intake, and feed efficiency

The results concerning the effect of different treatments on weight gain (WG), dry matter intake (DMI), feed efficiency (FE), and feed conversion ratio (FCR) of calves are presented in Table 3. The highest body weight gain, the highest dry matter feed efficiency, and the lowest FCR were associated with the NSM × SM interaction effect (*p* < 0.05).

**Table 3.** Effect of different treatments on weight gain, dry matter intake and efficiency, and feed conversion ratio in calves.

Treatments (% in diet)		Parameters			
NSM	SM	WG (kg)	DMI (kg)	FE (kg gain/kg DMI)	FCR (kg DMI/kg gain)
0	0	48.05^ab^	61.47^a^	0.78^b^	1.28^a^
5	0	48.20^ab^	56.88^b^	0.85^a^	1.18^b^
0	5	46.80^b^	57.61^ab^	0.81^a^	1.23^b^
2.50	2.50	51.73^a^	57.11^ab^	0.91^a^	1.10^b^
SEM		1.26	1.76	0.05	0.06
*p*-value					
NSM		0.000	0.000	0.000	0.000
SM		0.012	0.000	0.000	0.000
NSM × SM		0.000	0.000	0.000	0.000

*Different letters within a column indicate a statistically significant difference (*p* < 0.05) between treatment means for the traits. SEM = Standard Error of the Mean; WG: weight gain; DMI: dry matter intake; FE: feed efficiency; FCR: feed conversion ratio. Notes: 1. Dry matter intake includes intake from both fresh milk and solid feed. 2. Feed efficiency was obtained by dividing the average weight gain by the average dry matter intake. 3. Feed conversion ratio was obtained by dividing the average dry matter intake by the average weight gain.

The interaction effect of NSM × SM on weight gain and feed intake traits was significant, with the highest weight gain and feed efficiency and the lowest FCR, observed in this treatment (*p* < 0.05). *N. sativa* has a distinct aroma and a stronger, more pungent flavor compared to sesame, which may have influenced feed palatability and intake, potentially explaining the lower performance of the *Nigella*-alone treatment. These results indicate that neither NSM nor SM alone optimally increases calf weight, but their combined use improves performance. Findings from related studies suggest that a mixture of *N. sativa* and sesame has a synergistic effect, potentially leading to greater weight gain in calves. This effect is likely due to the combined action of active compounds in *N. sativa* (phenolic compounds like thymol and thymoquinone) and sesame (sesamin and sesamolin, lignans, and essential fatty acids), which simultaneously enhance nutrient digestion and absorption and improve energy and nutrient metabolism [10, 11, 14, 16, 26]. Compounds in *N. sativa* possess antioxidant, anti-inflammatory, and antimicrobial properties [14]. The lignans in sesame are also rich in antioxidant compounds that prevent oxidative stress [10, 12]. These compounds improve growth performance by enhancing nutrient digestion and absorption, increasing digestive enzyme activity, and reducing oxidative stress [11]. Consistent with these results, Obeidat et al. [2] also reported that incorporating sesame meal into small ruminant diets improved growth performance, average daily gain, and feed efficiency while reducing the cost per kg of gain, attributing this improvement to the positive nutritional effects of high-quality lipid and protein compounds in sesame meal. Furthermore, research with pre-weaning calves has shown that supplementing the starter diet with *N. sativa* meal increased body weight and improved the feed conversion ratio. This improvement was claimed to result from increased nutrient absorption and appetite stimulation in pre-weaning calves [8, 15].

Proper rumen development and accelerated function in pre-weaning calves are directly related to the quantity and quality of the starter diet. The consumption of starter feed, along with quality forage, is crucial due to its characteristics, such as palatability, high digestibility, stimulation of volatile fatty acid production, and its role in the development of digestive villi [3, 4, 29]. Results from various studies indicate that the use of highly digestible protein supplements is essential for pre-weaning calves during solid feed consumption due to insufficient rumen development, inadequate microbial protein production, and an underdeveloped gastrointestinal response, especially around weaning [1, 6, 30]. This may lead to deficiencies in essential amino acids and reduced calf growth [18, 20]. However, some reports are not consistent with the findings of the present study. Discrepancies in results may be due to differences in calf age and sex, varying rumen efficiency and development, differences in diet type and digestibility of the protein or phytogenic supplements used, or differing ratios of rumen-undegradable protein and, particularly, the varying digestibility of other dietary components [5, 19, 30, 31].

### 3.2. Biochemical blood parameters

The results on the effects of different treatments on the biochemical blood parameters of calves, including serum concentrations of glucose (Glc), triglyceride (TG), and total cholesterol (TC) at the end of the experimental period, are presented in Table 4. The *Nigella sativa* × sesame interaction effect was significant for all these parameters (*p* < 0.05).

**Table 4.** Effect of different treatments on biochemical blood parameters of calves at the end of the experimental period.

Treatments (% in diet)		Parameter (mg/dl)		
NSM	SM	Glc	TG	TC
0	0	80.17^a^	41.00^a^	127.13^a^
5	0	77.34^a^	34.34^b^	121.51^ab^
0	5	61.17^b^	32.17^b^	119.33^b^
2.50	2.50	65.00^b^	33.00^b^	108.85^c^
SEM		3.67	3.09	2.85
*p*-value				
NSM		0.000	0.000	0.005
SM		0.000	0.000	0.015
NSM × SM		0.042	0.012	0.000

*Different letters within a column indicate a statistically significant difference (*p* < 0.05) between treatment means for the traits. SEM = Standard Error of the Mean; Glc: Glucose; TG: Triglyceride; TC: total Cholesterol.

The lowest serum glucose levels were observed with the 5% NSM and mixed treatments, which were significantly lower than with the control and sesame-containing treatments. This reduction may indicate improved metabolic efficiency and optimal glucose utilization as an energy source under the influence of *N. sativa*’s active compounds. Compounds such as thymoquinone in *N. sativa* may reduce dependence on blood glucose by increasing insulin sensitivity or facilitating alternative metabolic pathways for energy production [4, 14]. A similar pattern was observed for changes in serum triglyceride and cholesterol concentrations, with all treatments containing NSM or SM, alone or combined, showing lower levels of these lipids than the control group. The lowest cholesterol level was specifically associated with the NSM × SM treatment. This significant reduction in blood lipids can be attributed to several potential mechanisms. Bioactive compounds in *N. sativa* (e.g., thymoquinone) possess hypolipidemic properties. Studies have shown that these compounds can reduce blood lipid concentrations by inhibiting cholesterol synthesis in the liver, increasing bile acid excretion, and stimulating fat breakdown (lipolysis) [2, 8, 9]. Additionally, the lignans in sesame (sesamin and sesamolin) are well-known as cholesterol- and triglyceride-lowering agents. They act by inhibiting intestinal cholesterol absorption and increasing fatty acid oxidation in the liver [17]. The synergistic and combined effect of these two meals is likely stronger than their independent effects. Their combination may lead to more potent inhibition of key enzymes in cholesterol synthesis, such as HMG-CoA reductase, and improve the lipid profile [13]. These results indicate that the use of *N. sativa* and sesame meals, alone or in combination, can enhance calves’ metabolic health by improving the blood lipid profile and reducing risk factors for metabolic diseases. Consistent with these findings, Abd El-Hack et al. [7] reported that adding *N. sativa* to poultry diets reduced blood cholesterol and triglyceride levels. Results from other animal studies showed that sesame lignans have strong hypocholesterolemic effects [10, 12, 13].

### 3.3. Humoral and cellular immunity and blood cell count

The results concerning the effect of different treatments on humoral and cellular immunity indices of calves at the end of the experimental period are presented in Table 5. The independent effects of NSM and SM were significant in most cases, and the NSM × SM interaction was significant for all traits (*p* < 0.05).

**Table 5.** Effect of different treatments on serum immunoglobulin concentrations and blood cell counts in calves at the end of the experimental period.

Treatments (% in diet)		Parameters				
NSM	SM	TP (gm/dl)	Alb (gm/dl)	IgG (gm/l)	RBC×10^6^	WBC×10^3^
0	0	5.25^b^	3.26^c^	5.13^c^	7.23^ab^	8.43^b^
5	0	5.55^ab^	3.63^b^	11.13^ab^	6.37^b^	7.75^b^
0	5	5.62^ab^	3.67^b^	13.13^a^	7.58^a^	10.65^a^
2.50	2.50	5.89^a^	3.97^a^	8.13^b^	6.13^b^	11.22^a^
SEM		0.21	0.29	1.54	0.48	1.39
*p*-value						
NSM		0.000	0.000	0.017	0.012	0.009
SM		0.012	0.000	0.008	0.001	0.003
NSM × SM		0.000	0.001	0.005	0.006	0.014

*Different letters within a column indicate a statistically significant difference (*p* < 0.05) between treatment means for the traits. SEM = Standard Error of the Mean; TP: Total Protein; Alb: Albumin; IgG: Immunoglobulin type G; RBC: Red blood cell; WBC: White blood cell.

Serum total protein reflects overall protein intake and balance, liver function, and hydration status. Serum total protein and albumin concentrations in all treatments were within the normal physiological range, indicating the absence of metabolic stress or liver damage. The slight but significant increase in total protein in the meal-supplemented treatments, particularly the mixed treatment, could be due to increased metabolic efficiency and feed and protein utilization in the calves. Bioactive compounds in *N. sativa* and sesame may have increased plasma transport protein concentrations by improving liver health and increasing the rate of conversion of dietary protein to body protein, thereby enabling more efficient use of protein for tissue synthesis. This aligns perfectly with the better weight gain in the mixed treatment (Table 3). Studies have shown that some feed additives can alter plasma protein concentration by improving protein metabolism while simultaneously improving growth indices [17, 31]. Albumin, which constitutes the majority of total protein, is primarily synthesized in the liver and plays a vital role in maintaining oncotic blood pressure and in transporting hormones, fatty acids, and other metabolites [2, 14]. The pattern of albumin changes was similar to that of total protein, with the highest concentration observed in the mixed treatment and the lowest in the control. The increase in total protein and albumin concentrations in the meal-receiving treatments, and the consequent improvement in growth and immunity indices, indicate an optimal allocation of protein resources within the body. These supplements appear to improve metabolism and digestion processes, leading to the direct use of amino acids in pathways for tissue protein synthesis (growth) and immunoglobulin synthesis (immune enhancement). This finding is consistent with the results of Obeidat et al. [11] on the positive effect of *N. sativa* compounds on body protein metabolism without inducing stress, as well as with the findings of Ardiana et al. [8] on improved immune response and reduced oxidative stress following the use of *N. sativa* compounds in animal diets.

The highest concentration of immunoglobulin IgG was observed with the 5% NSM, which was significantly higher (p < 0.05) than the other treatments and even better than the combined treatment. IgG, as one of the most important blood antibodies, plays a key role in humoral immunity (e.g., neutralizing pathogens and preventing infections) [17]. Its significant increase indicates a properly enhanced immune system. Active compounds in *N. sativa*, particularly thymoquinone, are known for their immunostimulatory properties. These compounds can enhance the immune response by stimulating B lymphocyte proliferation and increasing antibody production [8, 14].

Red blood cell (RBC) count was affected by the treatments, but the differences between groups were less pronounced. This parameter indicates oxygenation status and general health. The reported values appeared normal in all groups. White blood cell (WBC) count also increased significantly (*p* < 0.05), similar to RBCs. White blood cells are the soldiers of the immune system, and their increase indicates the innate immune system’s preparedness and activation to combat pathogens [22]. The anti-inflammatory and immune-modulatory properties of *N. sativa* may have induced an optimal immune response without causing excessive inflammation [8, 14, 15]. These results suggest that NSM has greater potential to enhance the immune system than SM. Although their combination also showed a significant improvement compared to the control group, NSM alone appears to be a stronger stimulus for antibody production. This immune-enhancing effect can be attributed to the antioxidant, anti-inflammatory, immune cell-boosting, and immune system-modulating properties of the phenolic and alkaloid compounds in *N. sativa*, which prevent oxidative stress and allow the body to focus its resources on optimal immune function [14, 15, 16, 17]. According to Min et al. [12], sesame meals are an ideal substitute for soybean meals in fattening beef cattle diets, reducing nitrogen excretion and strengthening the animals’ antioxidant defenses. Other studies have confirmed that dietary sesame meals increase IgG and IgM levels, enhancing both the immune response and the body’s antioxidant defenses [12, 13, 29, 30].

### 3.4. Health characteristics

The results for the effects of different treatments on the health characteristics of calves throughout the experimental period are shown in Table 6.

**Table 6.** Effect of different treatments on health characteristics of calves during the experimental period.

Treatments (% in diet)		Parameters				
**NSM**	**SM**	**IF**	**ID**	**FCS**	**IID**	**GHS**
0	0	23.36^a^	25.00^a^	1.83	18.33^a^	13.00^c^
5	0	15.00^b^	13.33^b^	1.92	15.00^b^	16.60^b^
0	5	7.67^c^	6.67^c^	2.06	13.67^b^	18.20^a^
2.50	2.50	10.00^c^	11.00^b^	2.08	8.33^c^	18.70^a^
SEM		2.45	2.37	0.13	2.85	0.92
*p*-value						
NSM	0.000	0.043	0.031	0.032	0.003	NSM
SM	0.011	0.004	0.054	0.017	0.000	SM
NSM × SM	0.000	0.007	0.033	0.001	0.005	NSM × SM

*Different letters within a column indicate a statistically significant difference (*p* < 0.05) between treatment means for the traits. SEM = Standard Error of the Mean; IF = incidence of fever %; ID = incidence of diarrhea %; FCS = fecal consistency score; IID = incidence of infectious diseases %; GHS = General health score.

All health traits were influenced by NSM and SM consumption, and the effects of the treatments on them were significant (*p* < 0.05). The dietary inclusion of NSM, SM, and their combination also improved health outcomes, as evidenced by reduced incidences of fever, diarrhea, and other infectious diseases and a consequent increase in the general health score.

The bioactive compounds in NSM and SM improve the health and performance of pre-weaning calves by strengthening the immune system, improving gut health, reducing oxidative stress, and increasing nutrient absorption [17, 27, 31]. These compounds are effective at inhibiting pathogens and reducing inflammation, particularly in preventing gastrointestinal diseases such as diarrhea and respiratory diseases such as pneumonia. The use of these materials in calf diets can be considered a natural strategy to improve growth performance, prevent various diseases, and enhance animal health [8, 14, 15].

Adding *Nigella sativa* meal to the diet reduces the prevalence of infectious diseases such as diarrhea and respiratory infections [9, 29]. These effects are due to the antimicrobial properties and immune-enhancing effects of thymoquinone, one of the active phytogenic compounds in *N. sativa* [9, 30]. Therefore, based on the obtained results, it can be stated that the active compounds in NSM and SM, such as thymoquinone, sesamin, antioxidants, and dietary fibers, influence the improvement of calf health traits through various mechanisms, including reducing inflammation, strengthening the immune system, improving digestion and absorption, and combating pathogens. The combined use of these two materials has shown better results due to synergistic effects [7, 9, 15, 31].

In this regard, Elsayed et al. [16] found that supplementing the diet of pre-weaning calves with black cumin seeds (*Nigella sativa*) improved blood hematology, metabolic responses, and immunity. The treatment also elevated levels of key blood antioxidants and inflammatory markers, which serve as indicators of oxidative stress and overall health. These events lead to improved health and reduced disease incidence. Also, Cardoso et al. [10], in their investigation, reported that dietary intake of sesame derivatives improved the blood lipid profile (e.g., reduced cholesterol and triglycerides) and increased total blood protein levels. Therefore, considering the results of the presented studies and the mode of action of the active substances in these two meals, it can be concluded that the antioxidant compounds in *N. sativa* and sesame improve animal metabolic health by neutralizing free radicals and reducing oxidative stress. These compounds also have positive effects on growth performance, health, and immunity indices and blood parameters of calves. By improving nutrient absorption, strengthening the immune system, and reducing oxidative stress, these compounds enhance the general health and performance of calves [8, 11, 14, 15].

## 4. Conclusions

Based on the comprehensive findings of this study, dietary incorporation of *Nigella sativa* meal (NSM) and sesame meal (SM) into the starter feed of pre-weaning Holstein calves has demonstrated substantial, multifaceted benefits. The research establishes that supplementing the diet with these phytogenic meals at 5% of dry matter, particularly in an equal NSM × SM mixture, significantly enhances growth performance, feed efficiency, and key health and immunity parameters. The synergistic interaction between the two meals was paramount, with the combined treatment yielding superior results, including higher body weight gain and greater improvement in feed efficiency compared to the control. This was accompanied by a remarkable optimization of the blood metabolite profile, evidenced by reduced glucose, triglycerides, and cholesterol, alongside significantly elevated levels of total protein, albumin, immunoglobulin G, and white blood cell count. These biochemical improvements underscore enhanced metabolic health and a more robust immune response, directly contributing to the calves’ overall vitality and disease resilience. Therefore, it is strongly recommended that livestock producers supplement calf starter rations with a 5% DM mixture of NSM and SM in equal proportions to harness these synergistic effects and achieve optimal productivity and herd health. As a practical recommendation, farmers should source high-quality, contaminant-free feed and ensure homogeneous mixing to guarantee consistent intake. However, certain limitations must be acknowledged. The availability and cost of these specific meals could be prohibitive in some regions, potentially limiting widespread adoption. Furthermore, the long-term effects of continuous supplementation beyond the pre-weaning period remain unknown and warrant further investigation. It is also crucial to consider potential variations in bioactive compound concentrations (e.g., thymoquinone and sesamin) across meal sources and processing methods, which could affect the consistency of the results. Despite these limitations, the evidence strongly supports the targeted use of NSM and SM as a natural and effective strategy to improve calf-rearing outcomes.

## Data Availability

The data presented in this study are available from the corresponding author upon reasonable request.

## List of abbreviations

NSM, *Nigella sativa* meal; SM, sesame meal; FE, feed efficiency; FCR, feed conversion rate; d, day; gm, gram; kg, kilogram; DM, dry matter; DMI, dry matter intake; ME, metabolizable energy; NEm, net energy for maintenance; NEg, net energy for growth; NDF, neutral detergent fiber; ADF, acid detergent fiber; EE, ether extract; TP, total protein; TG, triglyceride; TC, total cholesterol; AOAC, Association of Official Analytical Chemists; NRC, National Research Council; mm, millimeter; L, liter; dl, deciliter; ml, milliliter; mg/dl, milligram per deciliter; gm/l, gram per liter; CRD, completely randomized design; SEM, standard error of mean; *p*-value, probability value.
